# Knowledge of hepatitis C status moderates the relationship between history of drug treatment and sterile syringe use

**DOI:** 10.1371/journal.pone.0196157

**Published:** 2018-04-27

**Authors:** Kiva Ariani Fisher, Laura Michele Cahill, Stephanie Broyles, Marion Rorke, William Thomas Robinson

**Affiliations:** 1 Behavioral and Community Health Sciences, LSUHSC School of Public Health, New Orleans, Louisiana, United States of America; 2 Contextual Risk Factors, Pennington Biomedical Research Center, Baton Rouge, Louisiana, United States of America; 3 Community Health Division, Department of Public Health and Environment, City and County of Denver, Denver, Colorado, United States of America; 4 STD/HIV Program, Louisiana Office of Public Health, New Orleans, Louisiana, United States of America; University of Cyprus, CYPRUS

## Abstract

High-risk injection related behavior including use of non-sterile syringes is associated with negative health outcomes among people who inject drugs (PWID). Drug treatment programs have been reported to curb hepatitis C (HCV) transmission. This study aims to assess the role of drug treatment programs and knowledge of HCV status, and how they influence current injection-related risk. Data were collected in 2012 by the New Orleans arm of the CDC funded National HIV Behavioral Surveillance. Respondent driven sampling was used to recruit a sample of PWID. The analytic sample consisted of 473 participants. Univariate, bivariate, and linear regression analyses were performed. Findings indicated that history of drug treatment is associated with sterile syringe use among PWID. Further, knowledge of HCV status modifies the relationship between history of drug treatment and sterile syringe use in this sample. These findings highlight the importance of scaling up prevention efforts by expanding testing, counselling, and treatment for HCV among PWID who enter drug treatment facilities.

## Introduction

It is estimated that 4.6 million people in the United States are hepatitis C virus (HCV) antibody-positive [1]. Among people who inject drugs (PWID), rates of HCV range across age groups and have been reported as high as 90% among older injectors, and as low as 33% among younger injectors [2]. While sharing injection equipment (i.e. cookers, cotton, water) is associated with the transmission of blood-borne pathogens, sharing contaminated syringes has consistently been shown to carry the highest risk of infection and is in fact the principle mode of HCV transmission [3]. Therefore, the use of sterile syringes is an important public health prevention measure that can reduce HCV transmission among PWID [3].

Treatment for drug dependence is the prevailing gold standard for drug rehabilitation. While there is some evidence that supports the efficacy of drug treatment programs, other studies have found that drug treatment alone may be ineffective at reducing future drug using behavior. For example, previous meta-analyses have established the effectiveness of psychosocial treatment (i.e. cognitive behavioral and contingency management) for adults with illicit drug abuse or dependence, though the reported effect sizes were small to medium [4, 5]. Studies have also found that treatment through medication-assisted therapy [6] may reduce HIV transmission among PWID by 54% [7]. By contrast, other studies have determined that prevention programs such as drug treatment options may lack efficacy in curbing the transmission of HCV among PWID [8, 9]. Data from these studies suggest that as many as 80 percent of drug treatment recipients relapse into continued drug use [9]. Additionally, studies comparing the effectiveness of inpatient treatment with out-patient treatment programs have also yielded mixed results. These conflicting findings warrant further research on the success of treatment programs as a clear consensus on their effectiveness has not been reached. One explanation for this lack of consensus is that previous studies have not considered the role of current knowledge of disease status (i.e. HCV status awareness) and how it may influence the relationship between history of treatment and future risky injection practices.

Knowledge of HCV status has been found to influence injection sharing behavior [10]. For example, sero-sorting among PWID can drive decision-making around injection-related behaviors and influence risky injection practices that can facilitate the transmission of blood borne pathogens [11, 12]. Prior research has documented a positive association between self-reported HCV status and the HCV status of the last injection equipment sharing partner [13]. Given this association, it is surprising that few studies have investigated the role of HCV status awareness on the efficacy of drug treatment programs.

One study among individuals currently in drug treatment programs compared risky behaviors among participants reporting positive HCV infection with those who reported negative or unknown HCV status. Findings showed that HCV awareness was associated with increased recent syringe sharing compared with those with a negative or unknown HCV status [14] While this study explored the relationship between HCV awareness and injection risk among those presenting for drug treatment, it is unknown if this relationship operates differently among those with a past or history of drug treatment. As such, the literature has yet to converge on the efficacy of treatment programs and their influence on future behaviors that facilitate HCV transmission. Further, prior research has documented that using a sterile needle is a principle HCV prevention strategy [3, 15], therefore we chosen to focus solely on sterile needle use as our primary outcome.

This study aims to contribute to the body of literature by examining the moderating role of knowledge of HCV status on history of drug treatment and current injection use. Specifically, this study examines whether history of drug treatment is related to sterile syringe use while considering the influence of HCV status awareness.

## Methods

### Recruitment

In 2003, the Centers for Disease Control and Prevention (CDC) launched the National HIV Behavioral Surveillance (NHBS) to understand behavioral aspects of HIV transmission among three high-risk populations: men who have sex with men, injection drug users (i.e., PWID), and heterosexuals at increased risk for HIV infection. Twenty-one participating U.S. cities follow a well-established methodology and specific protocols disseminated by the CDC described in detail elsewhere [16]. Respondent driven sampling (RDS) [17] was used to gather a sample of PWID in New Orleans. This sampling methodology uses a modified snowball sampling technique with a coupon system (or chain-referral method) to produce a network driven sample of hard-to-reach communities, such as PWID [17,18]. The following study will focus on cross-sectional data collected in 2012. The data collected for this research was approved by the CDC, DHH, and LSUHSC IRBs. All data were collected anonymously.

### Participants

Eligibility criteria for this study were that participants had to live in the New Orleans metropolitan statistical area, be 18-years of age or older, and have injected non-prescription or illicit drugs in the past 12 months [19]. A total of 495 participants were eligible and completed the interview. Among the cases eligible for analysis, 22 (4.4%) had missing data for one or more of the variables of interest, yielding a final analytic sample of 473 cases.

### Instruments

The primary outcome, frequency of injection with a sterile syringe, was asked in the following way, “In the past 12 months when you injected, how often did you use a new, sterile needle? By a new, sterile needle, I mean a needle never used before by anyone, even you.” Participants responded using Likert scale options from never, rarely, about half the time, most of the time, or always. Items were coded along a five-point scale where higher values reflect increased risk behavior (i.e., zero indicates always using a new, sterile syringe and four indicates never using a new, sterile syringe in the past 12 months). Participation in drug treatment programs was assessed with the question: “Have you ever participated in a drug treatment program?” Previous knowledge of HCV status was assessed by asking, “Has a doctor, nurse, or other healthcare provider ever told you that you had hepatitis?” For those who answered yes, they were prompted to indicate which type of hepatitis they had. HCV status was confirmed by screening for HCV antibodies using OraQuick HCV Rapid Anitbody Test (OraSure Technologies, Inc.).

### Statistical analysis

Descriptive statistics provide information on participant characteristics for the analytic sample. Bivariate analyses were utilized to review pairwise associations with treatment history and HCV status. A linear regression model was created to examine the associations between use of sterile syringes and the predictors (i.e., history of drug treatment and knowledge of HCV status) after controlling for the following covariates: gender, race, network size, having ever been homeless, incarceration within the past 12 months, and number of years injecting. A cumulative logit model was also tested as a sensitivity analysis using the same set of predictors because the Likert scale outcome for sterile syringe use could be considered ordinal. The interaction effect was assessed by including the cross product term between knowledge of HCV status and history of treatment. Two additional *a priori* covariates for age and income were eliminated from analysis due to high levels of multicollinearity introduced by their strong relationship with years of injection and race in this sample, respectively.

## Results

Of the 473 participants that were included in the analytic dataset, 53.9% were African American, 80.6% were male, 37.0% were 40 years old or younger, and 60.8% reported heroin as the drug they injected most frequently (Table 1). Thirty eight percent of the sample population had never participated in a drug treatment program. Significant differences were found in treatment history for gender, age, frequency of injection, and type of drug most injected.

**Table 1:** Unadjusted sample statistics among PWID in New Orleans by knowledge of HCV status and history of drug treatment.

Participant Characteristic	Overall	Knowledge of HCV status			History of Treatment		
	(n = 473)%	Previous unknown or negative (n = 356) %	Previous positive (n = 117) %	p-value	Never treatment (n = 180) %	History of treatment (n = 293) %	p-value
Male	80.6	81.2	78.6	0.5459	75.6	83.6	**0.0315**
Black or African American	53.9	53.4	55.6	0.6809	47.1	58.0	**0.0222**
≤40 years old	37.0	39.3	29.9	0.0674	37.8	36.5	0.7830
Ever Homeless	69.6	69.4	70.1	0.8859	71.1	68.6	0.5646
Less than $10,000 household income	66.4	63.8	74.4	**0.0356**	65.0	67.2	0.6173
Injection Frequency				0.7321			**0.0119**
Less than once a week	18.2	18.8	16.2		23.3	15.0	
Between 1–7 days	27.5	27.8	26.5		30.6	25.6	
At least once a day	54.3	53.4	57.3		46.1	59.4	
Drug injected most frequently				**0.0025**			**0.0222**
Heroin	60.8	61.7	58.1		53.9	65.1	
Cocaine	21.4	19.7	26.5		26.7	18.1	
Speedball	9.1	7.6	13.7		7.8	9.9	
Other	8.7	11.0	1.7		11.7	6.9	
Network size				0.5687			0.2593
0–5	15.6	14.6	18.8		19.4	13.3	
6–20	50.5	51.7	47.0		50.6	50.5	
21–50	23.7	23.0	25.6		20.6	25.6	
50+	10.2	10.7	8.6		9.4	10.6	
Number of years injecting				**0.0075**			0.2445
0–5 years	17.3	19.4	11.1		21.7	14.7	
6–10	10.4	11.2	7.7		8.9	11.3	
11–15	13.7	14.6	11.1		13.9	13.7	
16–20	8.0	8.7	6.0		8.3	7.9	
21–25	10.4	11.0	8.6		12.2	9.2	
>25	40.2	35.1	55.6		35.0	43.3	
Held in a detention center, jail, or prison in the past 12 months	50.2	50.2	50.1	0.9571	60.0	49.8	0.8164

A multivariable regression model tested the effect of knowledge of HCV status and history of drug treatment on sterile syringe use after adjusting for gender, race, network size, homelessness, incarceration within the past 12 months, and number of years injecting. Additionally, the interaction between knowledge of HCV status and history of drug treatment was included in the full model. In this model, history of treatment was positively associated with sterile syringe use (*β* = 0.68, *SE* = 0.24, *p* = 0.0043) (Table 2). Further, the interaction effect for knowledge of HCV status and history of drug treatment (*β* = -0.60, *SE* = 0.27, *p* = 0.0240) was significant. A cumulative logit model was also tested to examine if treating sterile syringe use as an ordinal variable would influence the results from the linear model. The results from the logit model confirm that treatment was associated with sterile syringe use (*β* = 0.33, *SE* = 0.11, *p* = 0.0047) and there was an interaction effect for knowledge of HCV status and history of drug treatment (*β* = -0.28, *SE* = 0.12, *p* = 0.0233).

**Table 2:** Results from a regression model of drug treatment and knowledge of HCV status on sterile syringe use.

Variables	β-coefficient	SE	p-value
Drug treatment			
History of treatment	**0.68**	**0.24**	**0.0043**
Never treatment	(ref.)	—	—
Knowledge of HCV status			
Previous unknown or negative for HCV	0.35	0.14	0.2721
Previous positive for HCV	(ref.)	—	—
Gender			
Male	**0.40**	**0.13**	**0.0033**
Other	(ref.)	—	—
Ethnicity			
African American	**0.28**	**0.11**	**0.0090**
Other	(ref.)	—	—
Network size			
0–5	-0.33	0.20	0.1043
6–20	-0.06	0.17	0.7391
21–50	-0.26	0.18	0.1535
50+	(ref.)	—	—
Incarceration			
Not incarceration in the past 12 months	0.08	0.11	0.4741
Incarceration in the past 12 months	(ref.)	—	—
Homeless			
Never homeless	0.05	0.11	0.6580
Ever homeless	(ref.)	—	—
Number of years injecting	0.01	0.00	0.1092
Treatment history x Knowledge of HCV status	**-0.60**	**0.27**	**0.0240**

Adjusted general linear model with least square mean results corroborate the interaction effect for HCV knowledge and history of treatment for sterile syringe use. Individuals who had a history of treatment and self-reported positive for HCV were the least likely to use a sterile syringe (${\bar{x}}_{\mathrm{adj}}$ = 1.66, 95% CI 1.41–1.9). In contrast, those who had no history of treatment and self-reported positive for HCV were the most likely to use a sterile syringe (${\bar{x}}_{\mathrm{adj}}$ = 0.98, 95% CI 0.55–1.39). Significant differences between all groups means were observed at *p* = <0.0001.

## Discussion

The results of this study indicate that knowledge of HCV status differentially influences the relationship between treatment history and current injection risk behaviors. These findings demonstrate that history of treatment is associated with high-risk injection-related behaviors especially among PWID who already know they are HCV positive. In other words, HCV positive individuals with a history of treatment were less likely to use sterile syringes after adjusting for gender, race, network size, homelessness, incarceration in the past 12 months, and number of years injecting. This suggests a moderating effect of knowledge of HCV status on the relationship between treatment history and sterile syringe use.

While few studies have considered the influence of HCV status and history of drug treatment programs on current injection use, our study begins to close this gap in the literature [14]. Research suggests that among those enrolled in drug treatment programs, individuals who inject drugs are the highest risk patients [20]. They are more likely to engage in risky injection-related practices with over 60 percent reporting sharing syringes and other injection-related equipment [20, 21]. Their elevated risk could potentially be due to higher rates of social marginalization as well as the chronic nature of dependence, which alters the structure of the brain and increases the risk of relapse after treatment [21, 22]. This demonstrates the need for improved drug treatment tailoring. These findings support existing research, which suggest that knowledge of HCV status is an important psychosocial element to consider when addressing high-risk injection-related behaviors among PWID [10–12]. The majority of participants in our sample who had a history of treatment were aware of their HCV status. However, knowledge of one’s HCV status may not necessarily result in a positive change in injection-related behaviors. This is an important area for intervention and indicates a need for providers to educate clients on the implications of their HCV status and provide HCV treatment options such as direct acting antiviral therapy (DAA).

Through mathematical modeling and cost-benefit analyses, research suggests that targeting high-risk populations (e.g., PWID) with DAA’s is a cost-effective way to reduce the overall HCV prevalence [23–25]. Drug treatment programs could benefit by expanding HCV education, particularly among those who are HCV positive, increasing HCV treatment therapies (e.g., DAA’s), and providing psychological resources and coping mechanisms to buffer against potential negative consequences of relapse to prevent further transmission or acquisition of other blood borne pathogens (e.g. HIV). The high-risk injection behavior observed among HCV positive individuals with a history of treatment further highlights the importance of scaling up prevention efforts to identify and treat HCV. This could be accomplished by expanding HCV testing and counselling and initiating DAA therapies to PWID who enter drug treatment facilities. Our findings corroborate the need for targeted efforts and suggest that treatment facilities offer an important point of entry for linking PWID into HCV care. Further, reinfection rates among current PWID, after sustained virological response (SVR) is achieved, are low [26]. Thus, if PWID who enter a drug treatment facility are also treated for their HCV with DAA’s, and reach SVR, overall transmission rates will decline, should a future relapse occur.

This study has several limitations. First, while the sampling methodology, RDS, is becoming the gold standard for reaching hidden populations such as PWID, it operates via a peer-driven recruitment strategy [17], and subsequently may still miss PWID who are not connected to a network. Second, there are temporal limitations to consider regarding the HCV status and drug treatment variables. Specifically, the questions were not able to assess the timing of these events. Further, the cross-sectional design of the study does not allow us to determine causality in our findings. Third, the face-to-face interviews collected self-reported data that is subject to recall bias and social desirability bias, however the questionnaire was anonymous, which may have reduced this effect [27]. Additionally, our measure of drug treatment was non-specific, in other words, it is unknown whether the treatment type was in-patient, outpatient, residential, medication assisted treatment, or some other form of drug treatment program, which may influence risky injection practices differently [7,28].

While previous studies often conflate the use of sterile or new injection-related equipment [12], our study sought to determine the use of sterile needles, specifically, as the primary outcome. In this way, we could more accurately determine individual risk (e.g., for HCV, HIV). This study also expands our understanding of injection-related behaviors by obtaining information on recent injection-related risk behaviors (i.e., in the past 12 months) and the frequency of those behaviors. Since prior studies have only documented if people injected at all in the past month or past few months, this study provides a better picture of the actual frequency of recent risky injection behavior [29]. A final strength of this study is the large sample of PWID, which is more representative of the population and increases statistical precision.

In sum, these findings indicate that relying on drug treatment alone may not be sufficient to minimize risky injection practices associated with current injection-related behaviors but provides an important opportunity to mitigate future high-risk injection behaviors. Increasing drug treatment for HCV is recommended as the first line of defense and is considered to be a cost-effective way to treat PWID who already know they are HCV positive [21, 30]. Where DAA treatment is not available, other behavioral modification methods for strengthening traditional approaches and improving prevention strategies for PWID should be employed to reduce future risk. Healthcare workers should be aware of individual vulnerability factors, particularly among individuals who report knowledge of their HCV status, that contribute to sharing injection equipment. Future research on this topic should investigate risks and protective factors among PWID who have the resources and opportunity to enter drug treatment, and those who do not.
